# Postpneumonectomy transthoracic Esophagectomy – a case report: using anatomic change to create Extrathoracic Esophagogastric anastomosis

**DOI:** 10.1186/s13019-018-0742-5

**Published:** 2018-06-05

**Authors:** Qiuyuan Li, Jing Guo, Chenwei Li, Xinjian Li

**Affiliations:** 1 Department of Thoracic Surgery, Ningbo First Hospital, Ningbo, 315010 China; 2 Department of Thoracic Surgery, Shanghai Pulmonary Hospital Tongji University, Shanghai, China

**Keywords:** Pneumonectomy, Transthoracic esophagectomy, Esophageal cancer, Dissection, Esophagogastric anastomosis

## Abstract

**Background:**

Resection of primary esophageal cancer following previous pneumonectomy is a challenging procedure and was scarcely reported.

**Case presentation:**

Here we report a case in which reduced thoracic space was used in left transthoracic esophagectomy to counter the difficulties caused by previous left pneumonectomy.

**Conclusion:**

Retrograde dissection and infra-diaphragmatic esophagogastric anastomosis are examples of using postpneumonectomy changes to facilitate subsequent transthoracic esophagectomy for cancers of the lower esophagus.

## Background

The occurrence of primary esophageal cancer after preceding pneumonectomy for primary lung cancer is rare. For patients with previous pneumonectomy, transthoracic esophagectomy is always technically challenging given the postpneumonectomy anatomic deviations and a solitary lung as the remaining pulmonary reserve [1, 2]. We herein report a case of an adult patient who had a history of left pneumonectomy for lung cancer 12 years ago, and further received left transthoracic esophagectomy for a newly diagnosed esophageal cancer.

## Case presentation

A 72-year-old man came to our department with progressive dysphagia for nearly 2 months. At presentation, he could only take down fluid. The patient used tobacco and alcohol before he underwent left pneumonectomy for a pT2N0M0 primary squamous cell lung cancer 12 years ago, which was followed by 4 cycles of gemcitabine/carboplatin doublet chemotherapy. At postoperative follow-ups, he had been shown to be recurrence free.

**Fig. 1:**
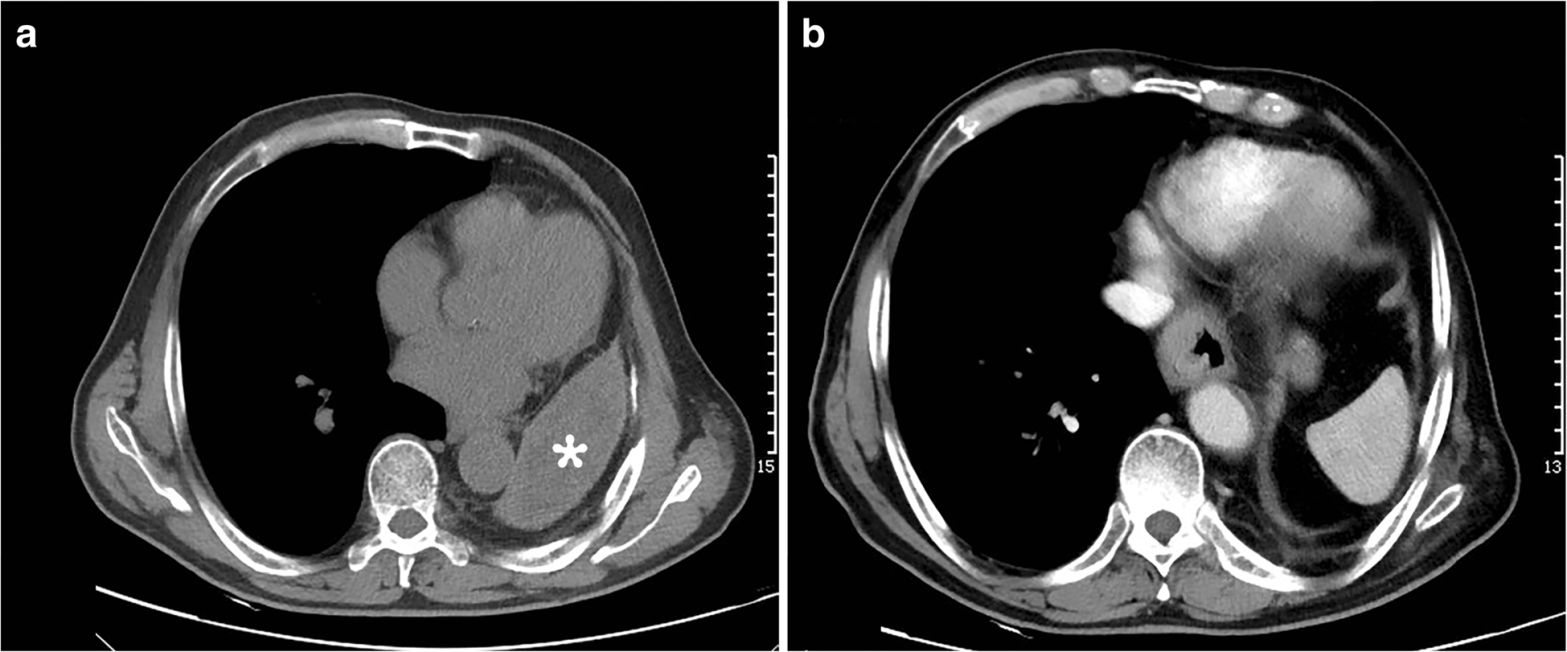
Preoperative computed tomography imaging of the patient showed typical postpneumonectomy changes, characterized by **a** hyperexpansion of the residual lung, mediastinal shift to the opacified postpneumonectomy space (asterisk) as well as **b** elevation of the hemidiaphragm superior to the level of the esophageal mass

Barium swallow and esophagogastroduodenoscopy were ordered and a distal esophageal mass was identified which was 36 cm from the incisors with extension to the cardia. Biopsy confirmed poorly differentiated adenocarcinoma. Computed tomography (CT) of the chest and abdomen demonstrated marked anatomic changes as a result of previous pneumonectomy, i.e. hyperexpansion of the right lung, mediastinal shift to the left hemithorax, elevation of the left hemidiaphragm and reduced left intrathoracic space with heterogeneous opacification (Fig. 1). No metastasis or lymphadenopathy was found after thorough examination including brain magnetic resonance imaging (MRI) and bone scan. Pulmonary function test showed a forced expiratory volume in one second (FEV1) of 0.98 L (46.6% of predicted) and a forced vital capacity (FVC) of 1.12 L (40.8% of predicted). No neoadjuvant treatment was given to the patient.

Based on the patient’s will and examination results, a left transthoracic esophagectomy with the ad hoc design of retrograde esophageal dissection and superior diaphragmatic reconstruction was pursuit for curative intent. This procedure was initiated with a regular posterolateral thoracotomy. Thoracic probing identified the imaging-proven anomalies that highly obscured normal anatomy. As such, retrograde dissection starting from the abdomen was justified.

**Fig. 2:**
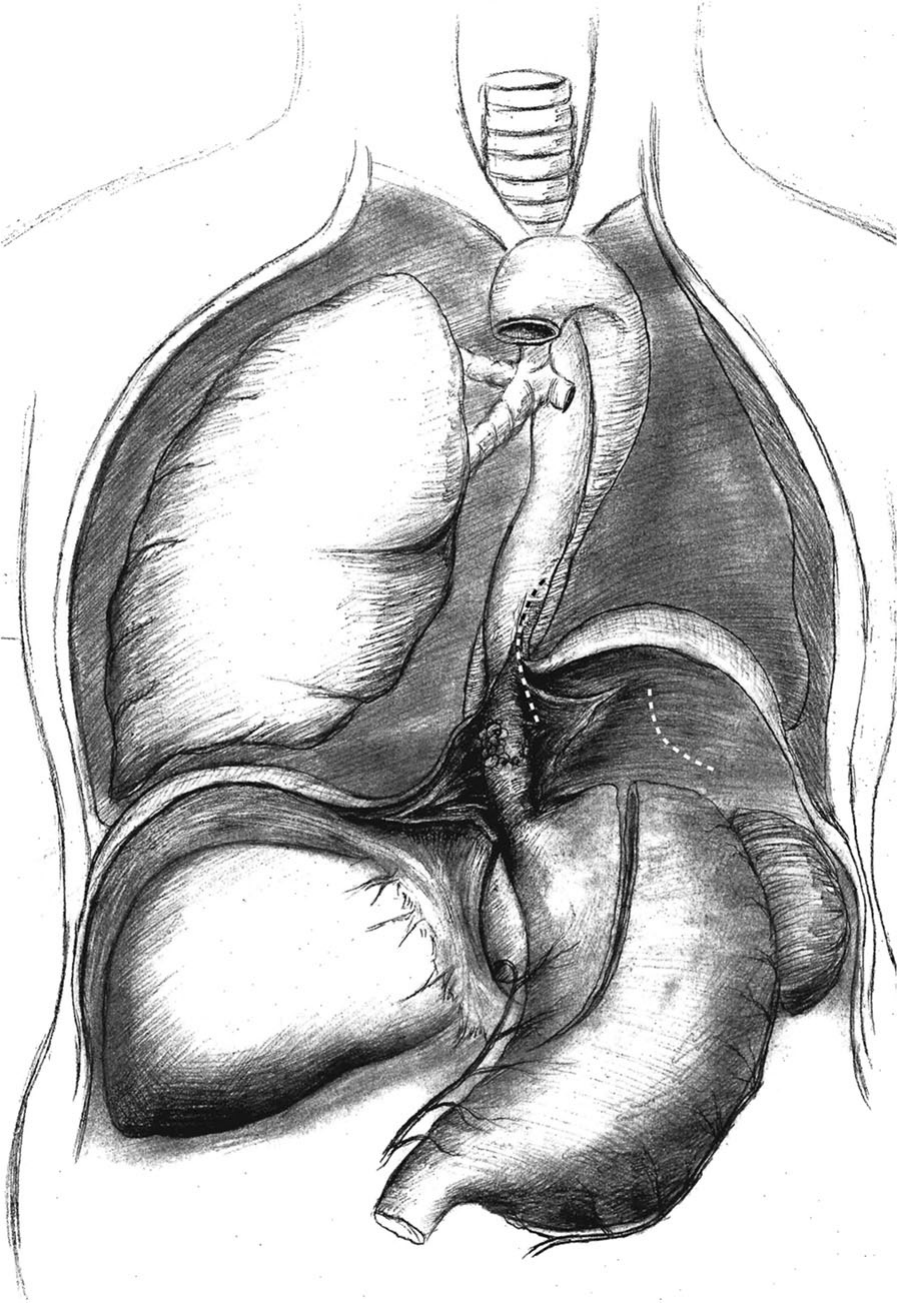
The retrograde dissection was initiated at the hiatus and carried cephalad after the abdominal operation was completed. The dashed lines denote the incision line of the diaphragm and the mediastinal pleura

This was achieved by the standard abdominal component of a typical left transthoracic esophagectomy (Fig. 2), which included exploration of the upper abdomen, gastric mobilization with vascular pedicles and lymph node dissection.

**Fig. 3:**
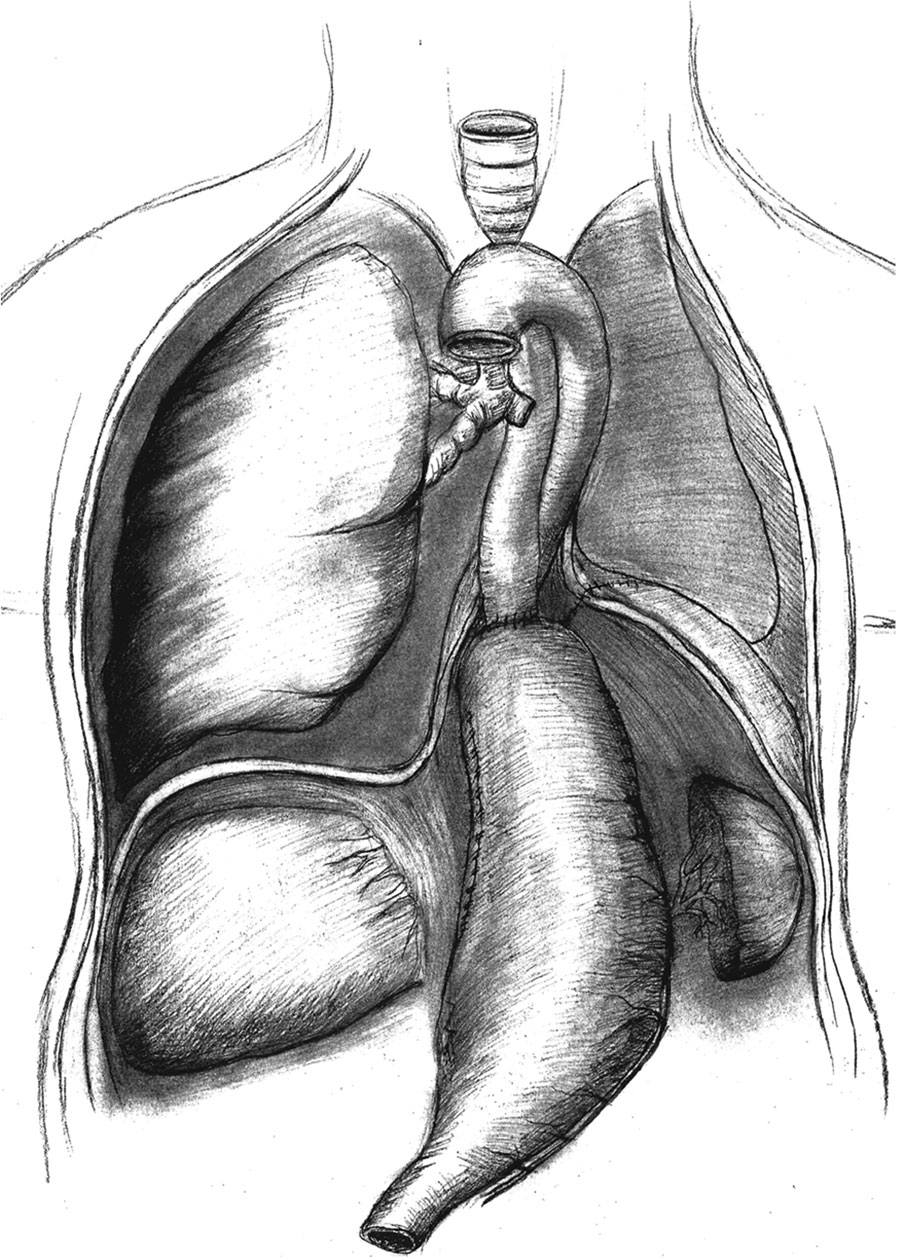
Reconstruction of the gastrointestinal tract with the diaphragm being anastomosed superior to its original position so that the esophagogastric anastomosis was left in the abdomen

Retrograde dissection of the esophagus was facilitated by the elevated diaphragm, which stretched and widened the esophageal hiatus. It was carried cephalad until 5 cm proximal to the esophageal mass, at which point the esophagus was transected. After the frozen section confirmed negative margin, the gastric conduit was prepared, and an end-to-end esophagogastric anastomosis was performed with an intraluminal circular stapler (Frankeman International Ltd., Suzhou, China), reinforced by several 4–0 absorbable sutures. An abdominal drainage tube was placed, and the diaphragmatic was reanastomosed superior to the esophagogastric anastomosis for an intentional precaution of postoperative anastomotic complications (Fig. 3). In the end, one chest tube was inserted, and decompression and nasojejunal feeding were initiated right after surgery.

In the postoperative course, ambulation was initiated on postoperative day (POD) 4. Oral feeding was restored on POD 7. Thoracic and abdominal drainage were terminated on POD 11 and POD 12 respectively after confirmation of anastomotic integrity. The patient was discharged on POD 14 with a normal chest CT. Postoperative pathology revealed a stage IIA primary adenosquamous esophageal carcinoma adventitia involvement (pT3N0M0).

The patient was followed up for 12 m, and was readmitted once for incision wound infection on POD 30, which required open drainage but no antibiotic use. No relapse was found.

## Discussion and conclusions

Esophagectomy in the setting of prior pneumonectomy is challenging and only few cases have been reported to date [1–5]. A dilemma remains regarding the approaching side, as pneumonectomy has left substantial deformity in the ipsilateral thoracic space, whereas operating on the healthy side puts the residual lung at stake. Several techniques including endobronchial blockers [4] and extracorporeal membrane oxygenation (ECMO) [5] have been introduced to enable surgery on the side contralateral to pneumonectomy. However, in the current case, we opted to enter the pneumonectized hemithorax and performed transthoracic esophagectomy with adaptations of retrograde esophageal dissection and infra-diaphragmatic anastomosis.

The objectives of this method were three-fold: first, to aid the dissection in the unfamiliar postpneumonectomy area under the guidance of improved vision from the abdominal side; second, to keep the contralateral hemithorax intact so as to minimize impact on the residual pulmonary reserve; third, to establish infra-diaphragmatic anastomosis to allow for improved management improved management for potential anastomotic leak. These are important as they circumvent the technical hurdles arising from the previous pneumonectomy while maintaining safety at a reasonable level. Also, this procedure distinguished itself by taking advantage of the existing anatomic abnormalities without additional use of dedicated devices, thus making it more affordable and less technology demanding.

As an alternative, transhiatal esophagectomy could be an approach that avoid thoracic entry. However, this approach was also compromised by postpneumonectomy changes, and risk was further added by previous mediastinal lymph node dissection. Albeit our treatment proved to be useful in this patient, the surgical approach should be optimized on an individual basis, and the procedure presented here is only applicable for tumor of the lower esophagus. The lesson we took from this case is that a mindset of out-of-the-box thinking should always be ready for various real-life clinical scenarios.

In summary, transthoracic esophagectomy post ipsilateral pneumonectomy is feasible, and safe dissection and extrathoracic esophagogastric anastomosis can be achieved by even harnessing postpneumonectomy changes. An example is retrograde dissection plus infra-diaphragmatic esophagogastric anastomosis, which facilitates transthoracic esophagectomy for cancers of the lower esophagus.

## Abbreviations

CT: Computed tomography
MRI: Magnetic resonance imaging
FEV1: Forced expiratory volume in one second
FVC: Forced vital capacity
POD: Postoperative day
ECMO: Extracorporeal membrane oxygenation
